# The potential effect of infliximab, dimethyl fumarate (DMF), and their combination in ciprofloxacin-induced renal toxicity in male rats

**DOI:** 10.25122/jml-2022-0197

**Published:** 2023-03

**Authors:** Saif Mohammed Hassan, Mahmood Jasim Jawad, Mohammed Ibrahim Rasool

**Affiliations:** 1 Department of Pharmacy, Al-Zahrawi University College, Karbala, Iraq; 2 Department of Pharmacology and Toxicology, College of Pharmacy, University of Kerbala, Kerbala, Iraq

**Keywords:** infliximab, DMF, nephrotoxicity, apoptosis, NF-κB

## Abstract

The aim of this study was to evaluate the effectiveness of infliximab and dimethyl fumarate (DMF) in reducing renal damage induced by ciprofloxacin. Forty rats were divided into five groups of eight each, with normal saline and CIP 600 mg IP administered to all animals in Groups 1 and 2 for ten days. Groups 3 and 4 were administered infliximab 7 mg/kg and DMF 30 mg/kg 24 hours before the CIP injections. Group 5 received a combination of infliximab/DMF after 24 hours of CIP. The levels of TNF-α, NF-Bp65, and IL-6 were measured, and the results showed that both infliximab and DMF had similar effects. However, the combination of infliximab and DMF had a robust anti-inflammatory and antiapoptotic impact, reducing TNF-α, NF-Bp65, IL-6, and Bcl-2 compared to the renal control group. Bcl-2 immuno-expression was lower in the ciprofloxacin group compared to the control group. DMF and infliximab had no effect on Bcl-2-positive cells, whereas infliximab increased the percentage of Bcl-2-positive cells substantially. CIP induced nephrotoxicity by increasing cytokine release and cell death signaling. Both infliximab and DMF are powerful TNF-α blockers that suppress cytokine release, preventing cell death and apoptosis caused by cytokines. Controlling inflammation and apoptosis can prevent nephrotoxicity.

## INTRODUCTION

Drug-induced nephrotoxicity is a serious concern and is often caused by oxidative stress and inflammation. Ciprofloxacin (CIP), a Fluoroquinolone antibiotic, has been linked to acute renal damage [1]. Infliximab, an anti-TNF-α medication, inhibits the production of proinflammatory cytokines by binding to TNF-α in the body. Dimethyl fumarate (DMF) is another medication that inhibits NF-kB by activating IKKb at Cys-179. This leads to the suppression of pro-inflammatory signaling pathways and reduced cytokine production. Fluoroquinolones, such as ciprofloxacin, inhibit bacterial DNA replication by targeting DNA gyrase, a type II topoisomerase, and topoisomerase IV. However, these antibiotics have been associated with various adverse effects, including heart arrhythmia, arthropathy, hypersensitivity reactions, and central nervous system effects such as agitation and insomnia [2,3].

Additionally, fluoroquinolones have been linked to acute kidney injury, with most case reports of nephrotoxicity involving ciprofloxacin [4,5]. Ciprofloxacin is a widely used antibiotic given to teenagers and adults [6]. Therapeutic levels of ciprofloxacin are generally safe, but excessive doses have resulted in acute renal damage [6,7]. This is believed to be caused by oxidative stress and inflammation, which are key pathways in drug-induced nephrotoxicity [8,9].

Several studies have shown that infliximab also reduces the production of other proinflammatory cytokines, lowering cell infiltration and acting as an effective anti-inflammatory agent [10,11]. DMF is a recently FDA-approved medication for multiple sclerosis (MS) and psoriasis [12]. It works by inhibiting the NF-κB pathway through binding and activating NF-κB at Cys-179, which suppresses NF-B release from its cytoplasmic complex (NF-κB-IκB), thereby reducing pro-inflammatory signaling pathways. Additionally, DMF has been shown to suppress inflammation by activating Nrf2 and inhibiting NF-B activity [13]. This occurs by disrupting Keap1-Nrf2 complex cysteine residues, releasing Nrf2, and translocating it to the nucleus. DMF also inhibits NF-κB by activating IKK β at Cys-179, which blocks NF-κB from its cytoplasmic complex (NF-κB-IκB) and reduces pro-inflammatory signaling pathways [14].

The aim of this study was to investigate the potential role of infliximab, DMF, and their combination in ameliorating renal damage induced by ciprofloxacin.

## MATERIAL AND METHODS

### Chemicals

Ciprofloxacin hydrochloride was manufactured by the Samara Pharmaceutical Company in Samara, Iraq. DMF was purchased from Sigma-Aldrich Company (U.S.A), while BCL-2, Caspase-3, TNF-alpha, and IL-6 were obtained from Merck Company (Germany). All additional substances and reagents utilized in this investigation were of analytical quality.

### Animals

Male Wistar rats weighing 200–240 g were obtained from the Animals Laboratory Research Center at Kufa University, Iraq. Rats were housed under standard laboratory conditions with a 12-hour light/dark cycle, adequate temperature and humidity, and ad libitum access to food and water. All experimental methods were carried out in accordance with the ethical protocols approved by the Committee of Animal Experimentation of Kufa University / College of Medicine, Kufa, Iraq, and the National Library of Medicine Guide for the Care and Use of Laboratory Animals (NIH Publication No. 85-23, revised 1996).

### Animal grouping and drug administration

In this study, 40 rats were randomly divided into five groups of eight rats. Group 1 received intraperitoneal injections (IP) of 0.9% NaCl and the solvent used to dissolve infliximab and ciprofloxacin. Group 2 was given intraperitoneal injections of ciprofloxacin (600 mg/kg body weight) twice daily for ten days [15]. Group 3 received infliximab 7 mg/kg 24 hours prior to the intraperitoneal injection of CIP, similar to Group 1 [15]. Group 1 was given 30 mg/kg of DMF 24 hours before the delivery of CIP, while Group 4 received an intraperitoneal injection of CIP. Group 5 received the same intraperitoneal injection of CIP as Group 1 and was administered infliximab plus DMF 24 hours beforehand [16,17]. All groups underwent intraperitoneal administration of ketamine hydrochloride at a dose of 50 mg/kg by Parke-Davis Eczacisi in Istanbul, Turkey. Kidney tissues were taken out and frozen until analysis [18].

### Homogenization of tissue

Kidney tissue samples were processed in phosphate-buffered saline under standard conditions and time. The resulting supernatant was stored at -80 C⁰ until further analysis. A protein assay of the tissue homogenate was performed using benzethonium chloride and quantified using a turbidimetric technique. The proteins were then measured at 404 nm using a turbidimetric assay (Architect c16000, Abbott Laboratories, IL, USA). The levels of TNF- α, NF- κB p65, interleukin-6, caspase-3, and Bcl-2 were determined using a commercially available TNF-α enzyme-linked immunosorbent assay kit (eBioscience, Vienna, Austria).

### Bcl-2-immunohistochemistry

Tissue sections were hydrated in 100% alcohol, followed by 70%, 50%, and distilled water for 3 minutes each. Antigen extraction was performed using Tris-EDTA, and sections were retrieved for 15 minutes using the Pathn Situ Multi-Epitope Retrieval System from Thermo Scientific. The sections were then cooled, rinsed in distilled water and PBS for 3 minutes, blocked for 10 minutes with Poly Excel H2O2, and washed in PBS. The tissue sections underwent immunohistochemical staining, involving incubation with a primary antibody for 45 minutes, followed by washing in PBS. Subsequently, the sections were incubated with Poly Excel Target Binder for 10 minutes, washed again in PBS, and incubated with a Poly Excel Poly HRP labeled polymer. Finally, the site of the antigen was visualized by staining with 3,3'-diaminobenzidine (DAB) substrate-chromogen [19]. Tissue slices were counterstained for 30 seconds with Mayer's hematoxylin, dried, and coverslipped. A negative control was included for each stain to ensure specificity. The thickest third of the MCC contained immune-positive and immune-negative cells. Positive cells in five randomly selected 400 fields per section were represented as a percentage. Poor immunoreactivity prohibited the quantification of terminal hypertrophic chondrocytes [20].

### Histopathological analysis

Samples from the kidney were fixed in 10 percent phosphate buffer formalin, dehydrated in alcohol, and embedded in paraffin for light microscopic studies. Hematoxylin and eosin stain (H&E) was used to stain five-micron tissue slices for general histopathological analysis. Light microscope observations were made on slides (Nikon Labophot, Japan).

### Statistical analysis

The mean ± standard error (SE) was used to express all results since our data followed a typical distribution. To compare multiple groups, we used a one-way analysis of variance (ANOVA), followed by Tukey's posthoc analysis when ANOVA showed a significant difference. The threshold for statistical significance was set at p ≤ 0.05.

## RESULTS

### Levels of NF-κBp65, IL-6, TNF, and cleaved caspase-3

**Figure 1:**
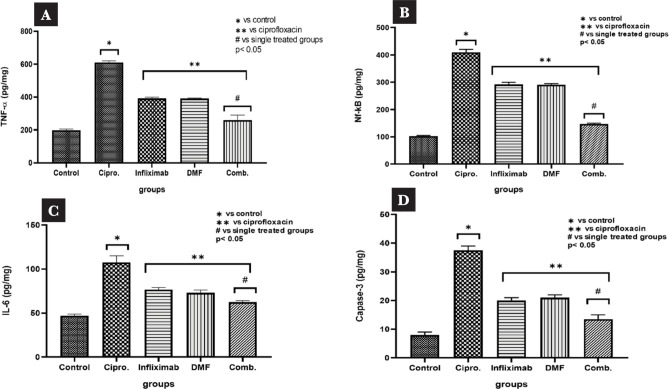
Renal levels of rats; A – TNF-α; B – NF-κBp65; C – IL-6 levels; and D – cleaved caspase-3 in response to treatment with infliximab, DMF, and their combination.

Kidney injury was associated with a significant increase in the levels of inflammatory cytokines, including TNF-α, NF-κBp65, and IL-6 (P ≤ 0.05) (Figure 1 A–D). Furthermore, the level of the apoptotic marker cleaved caspase-3 was significantly higher in the ciprofloxacin group compared to the control group (P ≤ 0.05), with negligible differences observed between the infliximab and DMF groups. Treatment with infliximab and DMF (7 mg/kg and 30 mg/kg of infliximab and DMF, respectively) significantly reduced the levels of TNF-α, NF-Bp65, IL-6, and cleaved caspase-3 (P ≤ 0.05) compared to the renal control group, suggesting a strong anti-inflammatory and antiapoptotic effect.

### Immunohistochemical study

**Figure 2:**
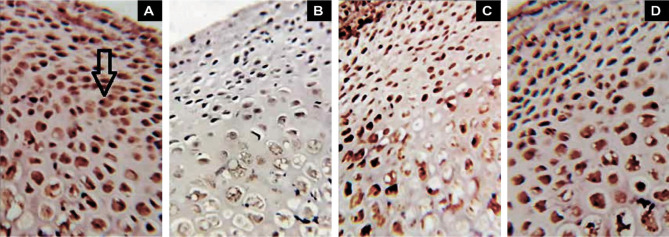
Bcl-2-immune stained of the control (A), ciprofloxacin (B), both infliximab & DMF (C) and combination (D) groups are shown in the images. The cytoplasm of the positive cells is stained brown (immunohistochemistry 400); arrow points to this

The Bcl-2 immunoreactivity was predominantly cytoplasmic in the control, infliximab, DMF, and combination groups (Figure 2 A–D). Statistical analysis revealed that the Bcl-2 immuno-expression in the ciprofloxacin group was significantly lower than in the control group (p=0.05). In contrast, the percentage of Bcl-2-positive cells increased significantly in all treatment groups compared to the ciprofloxacin group (p>0.05), although no significant differences were observed between the infliximab and DMF groups (Table 1).

**Table 1:** Immunohistochemical assessment of Bcl-2 expression in rat kidney tissue after treatment.

Groups	Bcl-2%	P-value
**Control**	71.5±2.43	0.05
**Ciprofloxacin**	8.16±2.54	0.05
**Infliximab**	45.33±5.18	0.32
**DMF**	43.16±2.54	0.30
**Combination**	53.33±5.15	0.05

## DISCUSSION

In this study, we investigated the effects of infliximab and DMF, alone and in combination, on kidney injury, inflammation, and apoptosis induced by ciprofloxacin in male Wistar rats. Ciprofloxacin is a broad-spectrum antibacterial drug belonging to Fluoroquinolones that is effective against a wide range of Gram-positive and Gram-negative microorganisms [21]. However, studies have shown that Fluoroquinolones, including ciprofloxacin, can generate reactive oxygen species that cause damage to kidney cells, leading to nephrotoxicity [7]. Our findings suggest that infliximab and DMF have a protective effect against the ciprofloxacin induced-renal injury model by decreasing TNF-α, NF-κBp65, and IL-6 in renal tissue, as well as upregulating Bcl-2 in the pretreated groups compared with the ciprofloxacin group. This protective effect is likely due to the inhibition of a number of pro-apoptotic and inflammatory cytokines that were produced in response to kidney injury [17,21].

Their activation is thought to be crucial for controlling the inflammatory response and cell death. Furthermore, it has been suggested that the phosphorylation and acetylation of the NF-κB p65 subunit are crucial for NF-κB activation [16, 22]. Our findings showed that renal tissue produced a significant amount of caspase 3, which results in kidney cell damage and apoptosis. Compared to ciprofloxacin-treated rats, there was a substantial decrease in caspase-3 levels and a significant increase in Bcl-2 levels.

Bcl-2 protein expression was critical in the regulation of apoptotic cell death and reduced the generation of free radicals and oxidative stress-induced cell death. Several studies have demonstrated the role of Bcl-2 in apoptosis control, as well as its ability to modulate pro-inflammatory cytokines, resulting in decreased inflammation [23,24]. The expression of Bcl-2 protein was considerably reduced in rats given ciprofloxacin, indicating that cellular damage could be caused by a drop in Bcl-2 expression. Infliximab and DMF treatment raised Bcl-2 expression, which was significantly elevated when both drugs were combined, and then reduced oxidative stress and inflammation. These therapeutic effects may be the basis for the protection against ciprofloxacin-induced pathological alterations in the liver and kidney of rats. We found that infliximab and DMF, alone and in combination, had renoprotective, anti-inflammatory, and anti-apoptotic effects primarily due to their ability to stabilize the inflammatory process, affect cell death signaling, and reduce pro-inflammatory mediators (TNF-α, NF-κBp65, and IL-6).

## CONCLUSION

Nephrotoxicity is caused by increased cytokine release and cell death signaling. Infliximab and DMF have been found to effectively suppress cytokine release and prevent cellular damage caused by cytokine-mediated inflammation and apoptosis production pathways, making them potent blockers of inflammation and apoptosis. Regulating inflammation and apoptotic mediators may protect against nephrotoxicity.
